# Mechanism of Acute Kidney Injury in Mild to Moderate Heat-related Illness

**DOI:** 10.14789/ejmj.JMJ24-0013-OA

**Published:** 2024-12-31

**Authors:** KENTA KONDO, NAOYUKI HASHIGUCHI, SHIN WATANABE, HIROFUMI NISHIO, YUJI TAKAZAWA, TOSHIAKI IBA

**Affiliations:** 1 Department of Emergency and Disaster Medicine, Juntendo University Graduate School of Medicine, Tokyo, Japan; 1 Department of Emergency and Disaster Medicine, Juntendo University Graduate School of Medicine, Tokyo, Japan; 2 Department of Sports Medicine, Juntendo University, Tokyo, Japan; 2 Department of Sports Medicine, Juntendo University, Tokyo, Japan

**Keywords:** heat-related illness, acute kidney injury, myoglobin, rhabdomyolysis

## Abstract

**Objectives:**

This study focuses on mild-to-moderate severity cases to examine the triggers initiating kidney injury.

**Materials:**

Patients aged ≥18 years with suspected heat-related illnesses at the Juntendo University Hospital Emergency and Primary Care Center between July and September 2020 and June and August 2022 were included.

**Methods:**

Blood samples were obtained during their visit, and the patients were categorized into two groups based on their cystatin-based estimated GFR (eGFRcys) values: a kidney injury group (eGFRcys < 60 mL/min/1.73 m^2^) and a non-kidney injury group (eGFRcys ≥ 60 mL/min/1.73 m^2^). Inflammation, coagulation, and skeletal muscle damage markers were compared between the groups, and markers related to the early development of kidney injury were examined.

**Results:**

Thirty-five patients were diagnosed with heat-related illnesses, and 10 were diagnosed with kidney injury. White blood cell count was higher in the kidney injury group (*P* < 0.01), whereas the levels of CRP and Interleukin-6 showed no significant difference between the groups. No statistically significant differences in coagulation markers were observed. In contrast, myoglobin, a marker of skeletal muscle damage, showed elevated levels in the kidney injury group (*r* = -0.80, *P* < 0.01) and demonstrated a stronger association with early kidney injury than creatine kinase (*r* = -0.38, *P* < 0.05).

**Conclusions:**

The predominant mechanism of acute kidney injury in mild to moderate heat-related illnesses appears to be tubular damage caused by myoglobin. Measuring myoglobin levels is essential to identify and exclude patients at risk of acute kidney injury due to heat-related illnesses.

## Introduction

Global warming has increased the frequency of natural disasters, including storms, floods, wildfires, and heat-related illnesses^1)^. Between 1991 and 2018, over one-third of all heat-related deaths were attributed to global warming^2)^.

Heat-related illnesses encompass various symptoms caused by hot environments. Increased risks of exertional and non-exertional heatstroke have stimulated active research. Severe heat stroke can cause central nervous system disorders, organ damage (including liver and kidney dysfunction), and blood coagulation disorders^3)^. Regarding the pathomechanisms of multi-organ damage, inflammation and coagulation play pivotal roles in its pathogenesis, aside from direct thermal injury^4, 5)^. Our previous studies demonstrated that heat injury could induce cytotoxic effects directly and indirectly through inflammation, resulting in non-programmed necrosis and programmed cell deaths. This process involves the activation of coagulation and progression to thromboinflammatory changes^6)^. Injured cells can release damage-associated molecular patterns (DAMPs), such as DNA, histones, and high-mobility group box 1 protein (HMGB1), further propagating systemic inflammation and coagulopathy^7)^.

Among the various organ dysfunctions, acute kidney injury (AKI) stands out as one of the most common organ disorders associated with heat-related illnesses^8)^. In recent years, sugarcane workers in Central America have exhibited a significantly high prevalence of chronic kidney disease (CKD) of unknown origin^9)^. Additionally, a retrospective population-based study of Taiwanese adults revealed a four-fold higher risk of CKD in patients with heat stroke^10)^. Regarding the development of CKD, studies project that repeated minor injuries can eventually result in irreversible renal damage^11)^. However, the precise mechanism of minor AKI induced by heat- related illnesses remains unclear. We hypothesized that inflammation and coagulation play major roles in the development of kidney injury in patients with heat-related illnesses. This study focused on the pathogenesis of AKI in mild-to-moderate heat- related illnesses, examining the factors involved in the development of kidney injury.

## Materials and Methods

### Patients and sample collections

This study, a prospective study, included patients aged ≥18 years suspected of having mild to moderate heat-related illness, seeking medical care at the Juntendo University Hospital Emergency and Primary Care Center between July and September 2020, as well as between June and August 2022. Mild to moderate heat-related illness in this study was defined as cases where the core body temperature did not rise above 40°C and central nervous system abnormalities were not present, thus excluding heat stroke. Following exposure to hot environments, these patients exhibited heat-related symptoms, including fever, dizziness, headache, fainting, nausea, malaise, weakness, and muscle cramps. Attending physicians in the emergency department conducted examinations, and blood samples were collected at the presentation. The exclusion criteria for this study comprised patients diagnosed with conditions other than heat-related illnesses or a history of CKD. For those with fever, routine tests were conducted to identify potential infectious foci, diagnosing infectious diseases upon detection of a focus.

### Ethics approval and consent to participate

This study was approved by the Ethics Committee of the Juntendo Institutional Review Board, Japan (Research ID H20-0071), and informed consent was obtained from all study participants. Patients who were older adults, under age 20, or unable to provide informed consent for other reasons obtained their consent through a proxy written by their next of kin. Certify that the study was performed in accordance with the ethical standards as laid down in the 1964 Declaration of Helsinki and its later amendments or comparable ethical standards.

### Measurements

The Kidney Disease Improving Global Outcomes (KDIGO) criteria were used for standardized assessment of common kidney disorders^12)^. However, due to the influence of muscle mass on creatinine levels and the limitations associated with urine volume, cystatin-based estimated GFR (eGFRcys) served as a substitute^13)^. Patients with eGFRcys < 60 mL/min/1.73 m^2^ were categorized into the kidney injury group, while those with eGFRcys ≥ 60 mL/min/1.73 m^2^ were in the non-kidney injury group. Various markers that may be associated with the development of AKI, such as inflammatory markers (white blood cell [WBC] count, C-reactive protein [CRP], ferritin, interleukin-6 [IL-6]), coagulation-related markers (platelets, D-dimer, prothrombin time-international normalized ratio [PT-INR]), and skeletal muscle damage markers (creatine phosphokinase [CK], myoglobin) were measured. Ferritin and IL-6 were measured using a Chemiluminescence Enzyme Immunoassay (CLEIA) and Electrochemiluminescence Immunoassay (ECLIA), respectively.

### Statistical analyses

The Kolmogorov-Smirnov test was performed to determine the normality of the data. The Mann-Whitney U test was used to compare medians between two groups of continuous variables, and Fisher's exact probability test was used to compare two groups of categorical data. Correlation coefficients were compared using Spearman's rank correlation coefficient. All *P*-values were two-sided, and those equal to or less than 0.05 were considered statistically significant.

All statistical analyses were performed using EZR (Saitama Medical Center, Jichi Medical University, Saitama, Japan), a graphical user interface for R (R Foundation for Statistical Computing, Vienna, Austria, version 4.0.0)^14)^. Specifically, EZR is a modified version of the R commander (version 1.42) designed to include statistical functions frequently used in biostatistics.

## Results

**Figure 1:**
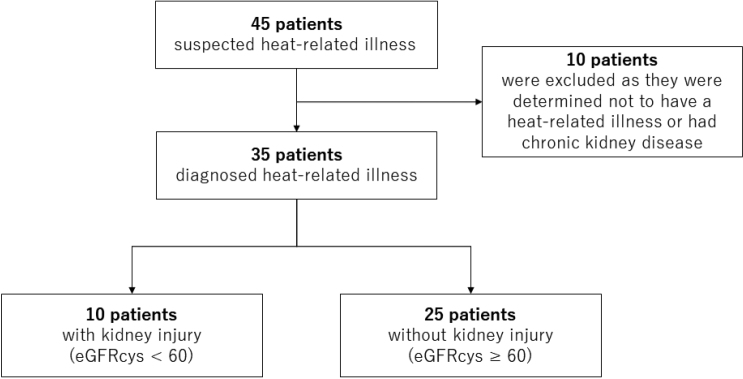
Patients selection The flowchart depicts patients’ selection and exclusion. A total of 45 patients presented with suspected heat-related illness and 10 patients who had different medical conditions or who had a history of chronic kidney disease were excluded. The patients were categorized into two groups: 10 cases with kidney injury (eGFRcys < 60 mL/min/1.73 m^2^) and 25 cases without. eGFRcys, Cystatin-based estimated glomerular filtration rate

From June to August 2020 and July to September 2022, 45 patients sought medical care for heat- related illnesses at the Emergency and Primary Care Center of Juntendo University Hospital. The patient eligibility, exclusion, and case counts are summarized in the flowchart (Figure 1). Of the 45 patients, 10 were excluded because of other diseases, such as infections or a history of CKD. The final analysis included 35 patients diagnosed with heat- related illnesses. Among them, 10 were identified as having kidney injury (eGFRcys < 60 mL/min/ 1.73 m^2^, kidney injury group), while 25 were identified as not having kidney injury (eGFRcys ≥ 60 mL/min/1.73 m^2^, non-kidney injury group).

Table 1 summarizes the characteristics and comorbidities of the patients diagnosed with heat-related illnesses. No significant differences in age or sex were observed between the kidney injury and non- kidney injury groups. Due to the difficulty in obtaining consent for measuring core body temperature, axillary temperature was measured instead. Cooling measures were applied in most cases before arrival, and the median body temperature was below 37.0℃ in both groups. Additionally, exertional heat-related illnesses were more common. Common symptoms included nausea, headache, dizziness, fainting, malaise, and muscle cramps. Since the study focused on patients with mild-to-moderate symptoms, none presented altered consciousness levels. No significant differences in comorbidities were observed.

**Figure 2:**
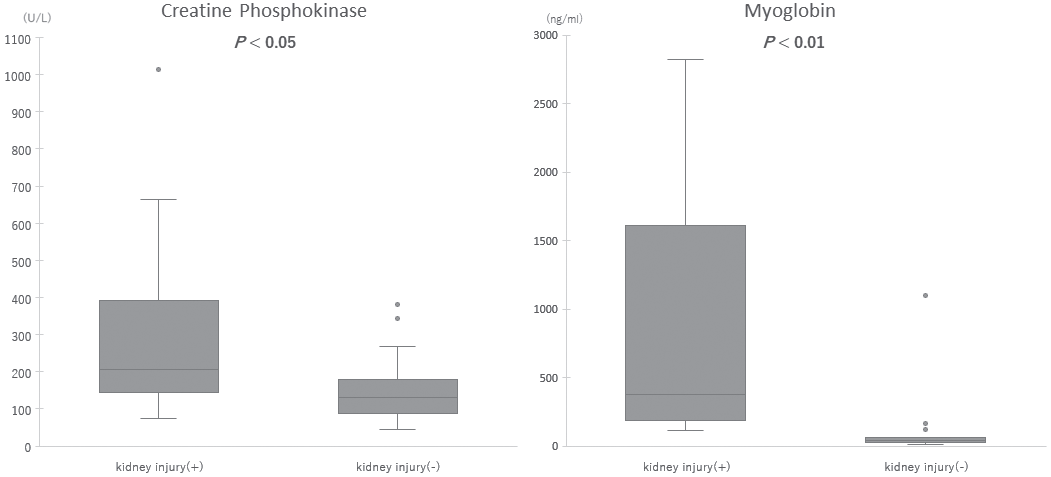
Comparison of the markers for rhabdomyolysis The creatine phosphokinase and myoglobin levels were compared between the kidney injury group (n=10) and the non-kidney injury group (n=25). The median values of both markers were higher in the kidney injury group. The box graphs reveal the upper, median, and lower quartile values of the markers for rhabdomyolysis. The whiskers represent the entire data range, specifically from the minimum to the maximum values. However, outliers, data points beyond this range, are not included in the whiskers. Outliers are defined as data points more than 1.5 times the interquartile range (IQR) away from the quartiles and are displayed as points on the boxplot.

**Figure 3:**
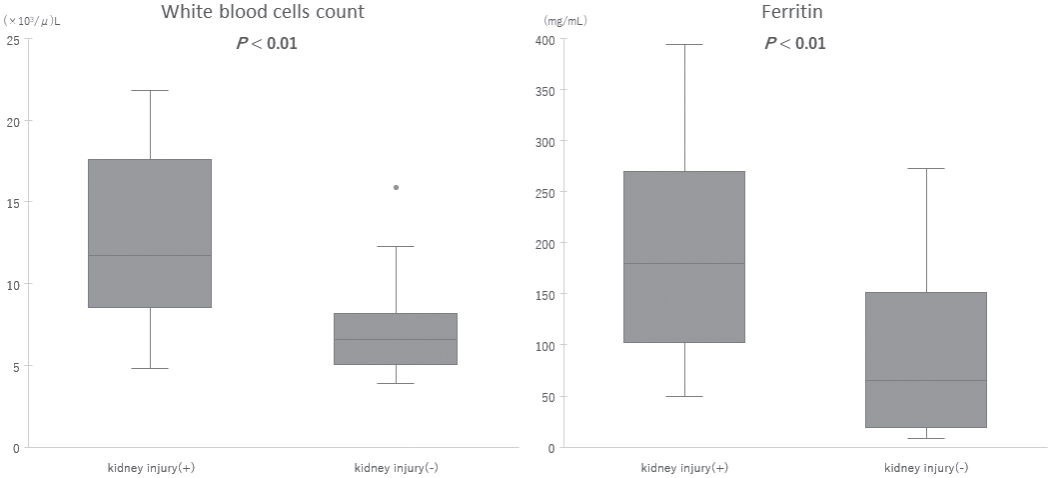
Comparison of the markers for inflammation The white blood cell count and ferritin levels were compared between the kidney injury group (n=10) and the non-kidney injury group (n=25). The median values of both markers were higher in the kidney injury group. The box graphs reveal the upper, median, and lower quartile values of the markers for inflammation. The whiskers represent the entire data range, specifically from the minimum to the maximum values. However, outliers, data points beyond this range, are not included in the whiskers. Outliers are defined as data points more than 1.5 times the interquartile range (IQR) away from the quartiles and are displayed as points on the boxplot.

**Figure 4:**
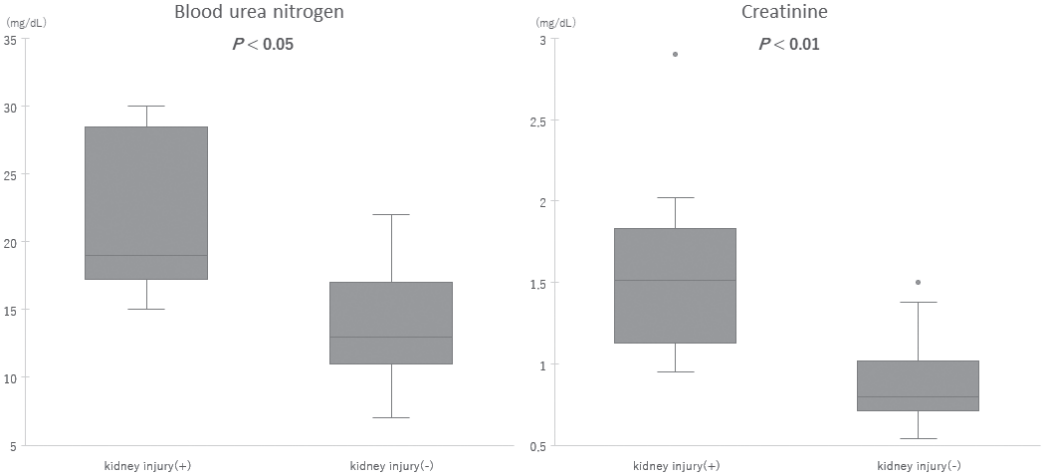
Comparison of the markers for renal function The blood urea nitrogen and creatinine levels were compared between the kidney injury group (n=10) and the non-kidney injury group (n=25). The median values of both markers were higher in the kidney injury group. The box graphs reveal the upper, median, and lower quartile values of the markers for kidney injury. The whiskers represent the entire data range, specifically from the minimum to the maximum values. However, outliers, data points beyond this range, are not included in the whiskers. Outliers are defined as data points more than 1.5 times the interquartile range (IQR) away from the quartiles and are displayed as points on the boxplot.

The median eGFRcys was 52.3 (43.7-57.5) mL/min/1.73 m^2^ in the kidney injury group, significantly lower than 92.1 (66.1-112.2) mL/min/1.73 m^2^ in the non-kidney injury group (*P* < 0.01). In the kidney injury group, eGFR was within the range of 43.7 and 57.5 mL/min/1.73 m^2^, indicating a mild-to-moderate kidney injury. The laboratory data during emergency room visits are summarized in Table 2. D-dimer levels below the upper detection range (1.0 μg/mL) and IL-6 levels below the upper detection range (1.5 pg/mL) were counted as 1.0 μg/mL and 1.5 pg/mL, respectively. Coagulation tests such as platelet count, D-dimer level, and PT-INR did not differ between the groups. Regarding the markers for skeletal muscle damage, both myoglobin and CK levels were higher in the kidney injury group (*P* < 0.05 and *P* < 0.01, respectively) (Figure 2). A correlation was observed between CK and eGFRcys (*r* = -0.38, *P* = <0.05), and a stronger correlation between myoglobin and eGFRcys (*r* = -0.80, *P* = <0.01). As for the inflammatory responses, the WBC count and ferritin levels were higher in the kidney injury group (*P* < 0.01, respectively) (Figure 3), whereas the levels of CRP and IL-6 were not significantly different between both groups. Renal damage markers, such as blood urea nitrogen (BUN), creatinine, and β2-microglobulin, were significantly higher in the kidney injury group (*P* < 0.05, < 0.01, respectively) (Figure 4). No significant differences were observed in Total Bilirubin (T-Bil), Aspartate Aminotransferase (AST), Alanine Aminotransferase (ALT), or electrolytes including sodium, potassium, and chloride.

**Figure 5:**
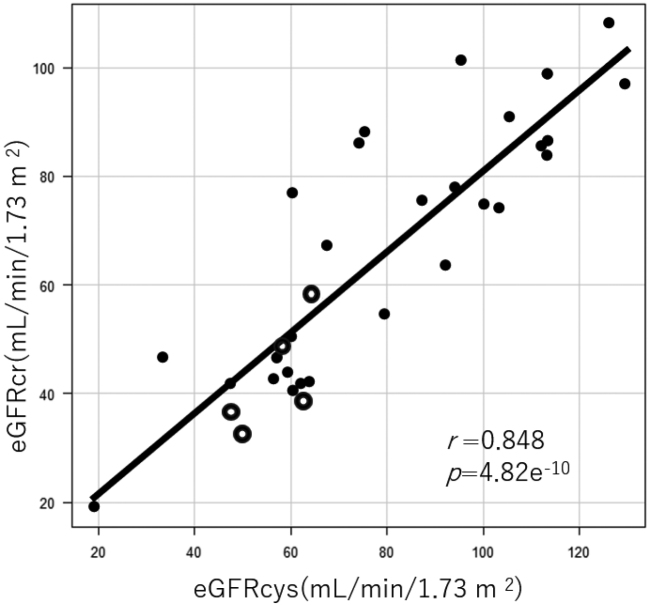
The correlation between eGFRcr and eGFRcys A significant correlation was recognized between eGFRcr and eGFRcys (*r* = 0.848, *p* = 4.82e-10). The black dots represent the data from samples with creatine phosphokinase ≤ 500 U/L and myoglobin levels of ≤ 1000 ng/mL, and the white dots represent the data from samples with creatine phosphokinase > 500 U/L or myoglobin levels of > 1000 ng/mL.

A significant correlation was observed between the creatinine-based eGFR (eGFRcr) and eGFRcys (*r* = 0.848, *P* = 4.82e^-10^) (Figure 5).

**Table 1:** Patient characteristics

Variable	Overall (n=35)	eGFRcys < 60 (n=10)	eGFRcys ≥ 60 (n=25)	*P*-value
Age (years)	45 (26-62)	59 (24-85.3)	44 (26.5-51)	0.31
Male sex, n (%)	26 (74.2)	9 (90.0)	17(68.0)	0.24
Body temperature (℃) (ranges)	36.8 (36.4-37.4)	36.9 (35.9-37.6)	36.8 (36.4-37.3)	0.71
GCS at arrival	15 (15-15)	15 (15-15)	15 (15-15)	1.00
Exertional heat-related illness, n (%)	31 (88.6)	8 (80.0)	22 (88.0)	0.61
Comorbidities, n (%)				
Hypertension	2 (5.7)	2 (20.0)	0 (0)	0.08
Hyperlipidemia	3 (8.6)	2 (20.0)	1 (4.0)	019
Hyperuricemia	4 (11.4)	2 (20.0)	2 (4.0)	0.56
Stroke	1 (2.9)	1 (10.0)	0 (0)	0.29
Coronary disease and/or heart failure	2 (5.7)	1 (10.0)	1 (4.0)	1.00

Unless otherwise specified, values are presented as median (IQR). eGFRcys, Cystatin-based estimated glomerular filtration rate; GCS, Glasgow Coma Scale

**Table 2:** Laboratory data

Variable	Overall (n=35)	eGFRcys < 60 (n=10)	eGFRcys ≥ 60 (n=25)	*P*-value
White blood cell count (×10^3^/μL) (3.9-9.7)	7.6 (5.5-11.0)	11.8 (8.5-17.6)	6.6 (5.1-8.2)	< 0.01
Hemoglobin (13.4-17.1)	14.4 (13.2-16.6)	16.5 (12.4-18.1)	14.3 (13.2-16.2)	0.30
Hematocrit (%) (40.4-51.1)	42.2 (38.7-47.5)	47.1 (36.9-51.4)	41.8 (38.8-46.5)	0.27
Platelet count (×10^4^/μL) (153-346)	237 (207-276)	259 (170-380.5)	237 (215.5-269)	0.84
T-Bil (mg/dL) (0.4-1.2)	0.89 (0.6-1.15)	1.10 (0.62-1.72)	0.77 (0.6-1.12)	0.08
AST (U/L) (5-37)	27.0 (19-34)	31.0 (27-32)	22.0 (18.5-35)	0.17
ALT (U/L) (6-43)	21.0 (15-31)	30.5 (19.3-36.3)	19.0 (14-29.5)	0.15
BUN (mg/dL) (9-21)	15.0 (13-19)	19.0 (17.3-28.5)	13.0 (11-17)	< 0.05
Cr (mg/dL) (0.6-1.0)	0.9 (0.74-1.35)	1.5 (1.13-1.83)	0.8 (0.71-1.02)	< 0.01
eGFRcr (mL/min/1.73 m^2^)	67.3 (42.7-86.4)	43.4 (35.1-47.0)	77.9 (60.7-93.9)	< 0.01
Cystatin C (mg/L) (0.63-0.95)	1.03 (0.79-1.24)	1.40 (1.25-1.68)	0.88 (0.78-1.07)	< 0.01
eGFRcys (mL/min/1.73 m^2^)	67.1 (58.1-101.7)	52.3 (43.7-57.5)	92.1 (66.1-112.2)	< 0.01
β_2_ microglobulin (mg/dL) (0.8-2.4)	1.9 (1.4-2.8)	2.8 (2.55-3.13)	1.5 (1.25-2.0)	< 0.01
CK (U/L) (57-240)	135 (92-231)	207 (145-393)	131 (88-180)	< 0.05
Myoglobin (ng/mL) (<149.9)	56.7 (31.5-252.3)	379.9 (185.0-1609)	39.2 (27.1-63.4)	< 0.01
CRP (mg/dL) (<0.29)	0.09 (0.04-0.16)	0.13 (0.06-0.21)	0.06 (0.04-0.12)	0.19
Ferritin (mg/mL) (39.4-340)	72.6 (33.2-180)	180.0 (103-270)	65.2 (18.9-151)	< 0.01
Interleukin-6 (pg/mL) (<7.0)	4.40 (2.07-13.8)	8.16 (4.15-14.08)	2.83 (1.89-16.25)	0.15
PT-INR (0.9-1-1)	1.00 (0.97-1.07)	1.00 (0.97-1.07)	1.02 (0.96-1.07)	0.99
D-dimer (μg/mL) (0-1)	1.2 (1.0-1.7)	1.6 (1.0-3.08)	1.2 (1.05-1.60)	0.33
Na (mmol/L) (135-145)	141 (139-143)	141 (135-144.3)	141 (139-142)	0.54
K (mmol/L) (3.5-5)	4.1 (3.8-4.4)	4.35 (4.08-4.65)	4.0 (3.75-4.2)	0.06
Cl (mmol/L) (96-107)	104 (98-105)	99 (92.8-106.3)	104 (101-105.5)	0.40
Uric acid (mg/dL) (3.5-6.9)	6.2 (5.0-7.8)	8.9 (6.1-10.8)	5.8 (4.9-6.6)	0.13

Unless otherwise specified, values are presented as median (IQR). T-Bil, total bilirubin; AST, aspartate aminotransferase; ALT, alanine aminotransferase; BUN, blood urea nitrogen; eGFRcr, creatinine-based estimated glomerular filtration rate; eGFRcys, cystatine-based estimated glomerular filtration rate; CK, creatine kinase; CRP, C-reactive protein; PT-INR, prothrombin time-international normalized ratio; Na, sodium; K, potassium; Cl, chlorine

## Discussion

This study aimed to explore the mechanisms underlying kidney injury in heat-related illnesses since we hypothesized recurrent minor kidney injuries can accumulate, resulting in the development of chronic kidney disease^11)^. Hence, we collected cases of mild to moderate severity to examine the triggers that initiate kidney injury.

AKI is a syndrome characterized by a rapid decline in renal function, and the presence of AKI is known to significantly worsen the prognosis of various diseases. The most common diagnostic criteria for AKI are the KDIGO guidelines, which include serum creatinine levels and urine output^15)^. However, urine output is not applicable because it cannot be measured during the patient's stay in the emergency room, and eGFR was utilized to diagnose AKI. Furthermore, urine output is significantly affected by dehydration, which is a common heat-related illness. Another concern is heat-induced skeletal muscle damage, which increases creatinine levels independent of kidney function^16)^. For these reasons, we used eGFRcys, which is less influenced by rhabdomyolysis^17)^. According to the KDIGO CKD guidelines, CKD is defined as the presence of markers of kidney damage or GFR < 60 mL/min/1.73 m^2^ (GFR categories G3a-G5) that persists for more than 3 months^12)^. An eGFRcr < 60 mL/min/1.73 m^2^ is significantly associated with an increased risk of all-cause mortality and death from cardiovascular diseases, and this is similar to eGFRcys^18)^. Therefore, we categorized the patients with eGFRcys < 60 mL/min/1.73 m^2^ as the kidney injury group. Although dehydration significantly affected GFR, no difference in hematocrit levels between the kidney injury and non-kidney injury groups was observed. Therefore, we believe that differences in this factor may not be substantial.

Heat stroke, the most severe stage of heat-related illnesses, has a high mortality rate and is complicated by multiple damages, such as central nervous system, kidney, liver, and hemostatic disorders. Inflammation and coagulation are assumed to be the two major factors contributing to adverse organ dysfunction in heat stroke, resembling the thromboinflammation observed in sepsis^7)^. In the pathogenesis of sepsis-associated AKI, not only increased inflammatory cytokines but also DAMPs released from injured host cells trigger an excessive immune response and damage tubular epithelial cells^19-21)^. Besides these factors, microthrombosis due to activated inflammation, coagulation, and vascular endothelial damage leads to microcirculatory disturbances and tissue ischemia^19, 20)^. We hypothesized that similar pathways are involved in the pathogenesis of AKI in heat-related illnesses. However, the results of the present study did not support this hypothesis. An increase in proinflammatory cytokines during heat stroke has been reported in both animal and clinical studies^4, 22)^. Although leukocytosis and ferritin levels were pronounced in the kidney injury group, no statistically significant difference in IL-6 levels was observed between both groups. Additionally, none of the coagulation markers showed statistically significant differences between them. The results suggest that the involvement of inflammation and coagulation is less evident at the beginning of kidney injury in heat-related illnesses.

In contrast, skeletal muscle damage was significant in the kidney injury group. Heat-related illnesses frequently result in severe rhabdomyolysis, releasing muscle cell contents, including electrolytes, myoglobin, CK, and other sarcoplasmic proteins^23)^. Myoglobin released from destroyed myocytes produces ferrous oxide (Fe^2+^), oxidized to ferric oxide (Fe^3+^), and eventually produces hydroxyl radicals^24)^. Thus, the massive release of myoglobin increases reactive oxygen species, which can cause renal tubular cell injury^7, 25)^. This study revealed significant differences between the kidney injury and the non- kidney injury groups concerning myoglobin and a significant increase in CK levels. The increase in myoglobin levels was more strongly associated with early kidney injury than with CK. This was likely because CK has no direct kidney-damaging effects. Therefore, evaluating myoglobin rather than CK levels for early kidney injury in heat-related illnesses may be more appropriate.

Creatinine measurement is widely accessible in most healthcare facilities. However, regarding the diagnosis of AKI in heat-related illnesses, an initial concern was that a creatinine-based diagnosis might be insufficient. Our study revealed a significant correlation between eGFRcys and eGFRcr. Even high CK levels of > 500 U/L or high myoglobin levels of > 1000 ng/mL did not deviate significantly from the trend line. Therefore, eGFRcr may serve as an indicator of kidney injury in clinical practice.

This study had a few limitations. First, it was conducted at a single institute with a limited sample size, thereby introducing an increased margin of error. Second, physicians in the emergency department diagnosed heat-related illnesses without the use of objective parameters. The accurate diagnosis of heat-related illnesses poses challenges, and it may not always be feasible to exclude other medical conditions. Additionally, there is a possibility that some patients with asymptomatic chronic kidney disease, who were unaware of their condition and had not sought medical care, were included in the study. The third limitation is the absence of urine sample investigation. Recent literature has suggested that urinary L-type fatty acid binding protein (L-FABP) is useful for detecting heatstroke-induced AKI in patients with severe heat- related illnesses requiring immediate treatment^26)^. The inclusion of urine samples in our study could have provided valuable insights. Additionally, the study focused exclusively on mild-to-moderate cases to investigate the early indicators of kidney injury. Subsequent studies should include severe cases to facilitate the observation of pathogenic progression from mild to severe AKI.

Myoglobin is more associated with early kidney injury than with inflammation or coagulation. The predominant mechanism of AKI in mild-to-moderate heat-related illnesses appears to involve renal tubular cell damage caused by myoglobin. Measuring myoglobin levels is ideal for excluding patients with heat-related illnesses susceptible to AKI, but measurement of creatinine can be the alternative.

## Funding

This work was supported in part by a Grant-in-Aid for Special Research in Subsidies for ordinary expenses of private schools from The Promotion and Mutual Aid Corporation for Private Schools of Japan.

## Author contributions

KK and TI researched the literature and conceived the study. KK, NH, and SW examined the patients and collected the specimens. KK wrote the first draft of the manuscript. HN and YT contributed to the editing of the manuscript. All the authors have read and approved the final version of the manuscript.

## Conflicts of interest statement

Toshiaki Iba participated on advisory boards of Japan Blood Products Organization, Asahi Kasei Pharmaceuticals, and Toray Medical. The other authors declare no competing interests. T. Iba, one of the Editorial Board members of JMJ was not involved in the peer review or decision-making process for this paper.
